# The time course of muscle-tendon properties and function responses of a five-minute static stretching exercise

**DOI:** 10.1080/17461391.2019.1580319

**Published:** 2019-03-01

**Authors:** A. Konrad, M. M. Reiner, S. Thaller, M. Tilp

**Affiliations:** Institute of Sports Science, Graz University, Graz, Austria

**Keywords:** Stiffness, ultrasound, passive resistive torque, maximum voluntary contraction, range of motion

## Abstract

The purpose of this study was to investigate the time course of the changes of muscle-tendon mechanical properties and the function responses of the plantar flexor muscles following 5 × 60 s of static stretching.

Fourteen healthy volunteers were tested on four separate days in a random order with three different rest times (0, 5, 10 min) after 5 × 60 s of stretching or following a control period without stretching. During each test, the dorsiflexion range of motion (RoM), passive resistive torque (PRT), and maximum voluntary contraction (MVC) were measured with a dynamometer. Ultrasonography of the gastrocnemius medialis (GM) muscle-tendon junction displacement and motion capture allowed us to determine the length changes in the tendon and muscle, respectively, and hence to calculate their stiffness.

We observed an increase in RoM and decrease in MVC at 0, 5, and 10 min post-stretching. This could be attributed to an increase in muscle elongation which lasted at least 10 min. A decrease in muscle-tendon stiffness was observed immediately, but not 5 or 10 min after the stretching. A decrease in PRT and muscle stiffness was observed up to 5 min after the stretching. No changes were detected in tendon stiffness or in any variable in the control group.

The effects of a 5 × 60 s static stretching exercise changes the muscle-tendon functions (RoM, MVC), which are related to mechanical changes of the muscle but not the tendon structure, respectively. Although the functional changes last for at least 10 min, changes in muscle stiffness were only observed up to 5 min after the stretching exercise.

## Highlights

- We observed an increase in RoM and decrease in MVC at 0 min, 5 min, and 10 min post-stretching.
- A decrease in PRT and muscle stiffness was observed at 0 min and 5 min but not at 10 min post-stretching.
- No changes were detected in tendon stiffness at any time point.

## Introduction

Stretching is generally divided into static, ballistic, and proprioceptive neuromuscular facilitation (PNF) stretching (Magnusson et al., 1996), and is commonly used in sports as a warm-up routine (McHugh & Cosgrave, 2010). With regard to static stretching, the increased range of motion (RoM) following a single stretching exercise goes along with a decrease in overall muscle-tendon stiffness (Kay, Husbands-Beasley, & Blazevich, 2015; Konrad, Budini, & Tilp, 2017; Konrad, Stafilidis, & Tilp, 2017) and passive resistive torque (PRT) (Konrad, Budini, et al., 2017; Konrad, Stafilidis, et al., 2017; Nakamura, Ikezoe, Takeno, & Ichihashi, 2013). Distinguishing between muscular and tendon structures of the muscle-tendon unit (MTU), there have been conflicting reports about the effects of acute static stretching. While Kay and Blazevich (2009), Kay et al. (2015), Konrad, Budini, et al. (2017), and Konrad, Stafilidis, et al. (2017) reported a decrease in stiffness of the muscle component, Kubo, Kanehisa, Kawakami, and Fukunaga (2001) and Kato, Kanehisa, Fukunaga, and Kawakami (2010) reported a decrease in active tendon stiffness (measured during active contraction) and passive tendon stiffness (measured when MTU is passively stretched) without changes in the muscle stiffness, respectively. These controversial results could be explained by the different stretching durations or intensities applied. While shorter stretch durations and lower stretch intensities are related to changes in muscle structure only, tendon structure seems to be only affected following longer stretch durations (>10 min) and/or greater stretch intensities (e.g. including maximum active contractions).

Similarly, stretch duration has also been reported to be related to the effects on performance. Kay and Blazevich (2012) showed in their review that only static stretching interventions that lasted longer than 60 s might have a disadvantageous effect on maximum performance output. However, until now, it is not clear how long a possible decrease in performance will last.

Mizuno and co-workers reported that an increase in RoM following a 5-min static stretch will last between 30 and 60 min (Mizuno, Matsumoto, & Umemura, 2013b). However, the decrease in muscle-tendon stiffness seems to recover within 5 min (Mizuno, Matsumoto, & Umemura, 2013a). This decrease in muscle-tendon stiffness was associated with an increased displacement of the muscle-tendon junction (an indication of increased muscle belly length) up to 5 min after stretching at 15° of dorsiflexion only (not at 5° or 10°). This was in accordance with the results of Kay and Blazevich (2009), who reported a decrease in muscle stiffness immediately after 3 min of stretching, which recovered 30 min after the stretching. Responses up to 30 min after the stretching were not investigated.

However, to the best of the authors’ knowledge, the precise time course of the response of muscle and tendon properties (e.g. passive muscle and tendon stiffness, active tendon stiffness) and function responses (e.g. RoM, maximum voluntary contraction (MVC)) within the first 10 min after stretching is not yet known.

Therefore, the objective of this study was to analyze the time course (immediately, 5, and 10 min after stretching) of the properties and function responses of the plantar flexor muscle-tendon system following a 5-min stretching exercise. We hypothesized an increase in RoM and a decrease in PRT and MVC, immediately, 5, and 10 min after stretching. We further assumed that these changes would be accompanied by a decrease in muscle and tendon stiffness and that these changes would last for 5 min, but would have recovered 10 min after the stretching.

## Material and methods

### Experimental design

On the first day subjects were familiarized with the lab equipment, with all assessments (RoM, passive, active), and the stretching regime. Moreover, Participants visited the laboratory for further four sessions on different days (with a 2–7 days break in between) at the same time of day in order to assess the effects of stretching immediately (0min_post), 5 min (5min_post), and 10 min (10min_post) after the stretching, as well as in a control (C) condition without stretching, in a randomized order. Before and after the four conditions (0, 5, 10 min, and C), the RoM, PRT, MVC torque, muscle-tendon stiffness, muscle stiffness, and passive and active tendon stiffness of the gastrocnemius medialis (GM) muscle were determined.

### Subjects

Seven healthy female (mean ± SD; 24.9 ± 3.1 years, 166.0 ± 6.1 cm, 60.0 ± 8.4 kg) and seven healthy male (mean ± SD; 27.5 ± 8.3 years, 180.1 ± 6.2 cm, 75.9 ± 6.5 kg) volunteers with no history of lower leg injuries participated in this study. Subjects were informed about the testing procedure, but were naive of the study's aim and hypotheses.

The study was approved by the local research ethics board and written informed consent was obtained from all volunteers before the onset of the experimental procedures.

### Measures

The temperature in the laboratory was kept constant at around 20.5°C. Measurements were performed without any warm-up and in the following order: pre-tests: RoM (1-min rest), PRT (1-min rest), MVC (1-min rest); intervention: stretching for 5 × 60 s; post-tests: immediately following stretching, or following 5 min of rest, or following 10 min of rest in the same order (RoM (1-min rest), PRT (1-min rest), MVC). In the control trial, the post-tests were performed without a prior stretch, 10 min after the pre-tests.

#### RoM measurement

RoM was determined with an isokinetic dynamometer (CON-TREX MJ, CMV AG, Duebendorf, Switzerland) in a seated position with a hip joint angle of 110°, with the foot resting on the dynamometer foot plate and the knee fully extended. Two oblique straps on the upper body and one strap around the thigh were used to secure the participant to the dynamometer and exclude any evasive movement. The foot was fixed barefooted with a strap to the dynamometer foot plate, and the estimated ankle joint centre was carefully aligned with the axis of the dynamometer to avoid any heel displacement. Participants were moved to the neutral ankle joint position in the dynamometer (90° between foot sole and tibia) and were subsequently asked to regulate the motor of the dynamometer with a remote control to get into a dorsiflexion (stretching) position until a maximum tolerable stretch was reached. The angular velocity of the dynamometer during this procedure was set to 5°/s. The difference between neutral position and the maximum dorsiflexion was defined as the dorsiflexion RoM.

#### Passive resistive torque (PRT) measurement

During this measurement, the dynamometer moved the ankle joint from a 20° plantar flexion to the individual end dorsiflexion RoM which was previously determined in the RoM measurement. During pilot measurements, we recognized a conditioning effect during the first two passive movements, similar to the active conditioning reported by Maganaris (2003). Therefore, the ankle joint was moved passively for three cycles and measurements were taken during the third cycle to minimize bias due to conditioning effects. Similar to the studies by Kubo, Kanehisa, and Fukunaga (2002) and Mahieu, Cools, De Wilde, Boon, and Witvrouw (2009), the velocity of the dynamometer was set to 5°/s to exclude any reflexive muscle activity. PRTs before and after the intervention were compared at the same angle of stretch (at the lower maximum RoM of pre- and post-stretching, respectively) to assess tissue resistance. Participants were asked to relax during the measurements.

#### Maximum voluntary contraction (MVC) measurement

MVC measurement was performed with the dynamometer at an ankle position of 10° of plantar flexion. Participants were instructed to perform two isometric MVCs of the plantar flexors for 5 s, with rest periods of at least 1 min between the measurements to avoid any fatigue. The attempt with the highest MVC torque value was taken for further analysis.

#### Electromyography (EMG)

Muscular activity was monitored by EMG (myon 320, myon AG, Zurich, Switzerland) during PRT and MVC measurements. After standard skin preparation, surface electrodes (Blue Sensor N, Ambu A/S, Ballerup, Denmark) were placed on the muscle bellies of the GM and the tibialis anterior according to SENIAM recommendations (Hermens et al., 1999). In the RoM and PRT measurements, the raw EMG was monitored online to ensure that the subject was relaxed. In the case of an increase in the EMG of the GM or the tibialis anterior being observed, the RoM or PRT measurement were repeated.

#### Measurement of elongation of the muscle-tendon structures

A real-time ultrasound apparatus (mylab 60, Esaote S.p.A., Genova, Italy) with a 10-cm B-mode linear-array probe (LA 923, Esaote S.p.A., Genova, Italy) was used to obtain longitudinal ultrasound images of the GM.

**Figure 1.**
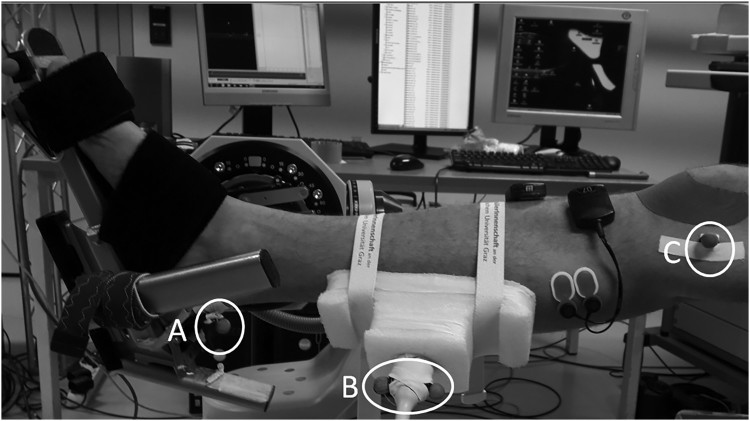
Experimental setup for the calculation of muscle and tendon lengths, with reflective markers on the calcaneus (A), two on the ultrasound probe (B), and on the medial epicondyle of the femur (C).

During the RoM, PRT and MVC measurements, the ultrasound probe was placed on the distal end of the GM (as described in a previous study, Konrad, Gad, and Tilp (2015), see Figure 1), where the muscle merges into the Achilles tendon, i.e. the muscle-tendon junction (Kato et al., 2010). The ultrasound probe was attached to the lower leg with a custom-built styrofoam block and secured with elastic bands to prevent any displacement of the probe. During a previous study (Konrad, Budini, et al., 2017; Stafilidis & Tilp, 2015), we confirmed that this kind of fixation of the ultrasound probe did not lead to any unwanted shifts of the probe during the measurement. To determine the muscle displacement during PRT and MVC measurements, the echoes of the muscle-tendon junction in the ultrasound videos were manually tracked (Kato et al., 2010).

The ultrasound images were recorded at 25 Hz. During PRT and MVC measurements, the videos were synchronized with the other data with a custom-built manual trigger. The videos were cut and digitized in VirtualDub open-source software (version 1.6.19, www.virtualdub.org) and analyzed in ImageJ open-source software (version 1.44p, National Institutes of Health, U.S.).

Each video was analyzed by two investigators, and the mean values of the measurements were used for further analysis of the muscle-tendon structure. Except for the principal investigator, the further investigator was neither informed of the hypotheses of the study nor the group allocation of the subjects. During the analysis of the PRT and RoM measurement every fifth frame was analyzed by the investigators, corresponding to a time resolution of 0.08 s. Moreover, during the analysis of the MVC measurement every second frame was analyzed, corresponding to a time resolution of 0.2 s.

#### Tendon and muscle lengths

Tendon and muscle lengths were analyzed during the PRT and MVC assessments, using a combination of ultrasound and 3D kinematics. Reflective markers were placed on the calcaneus (Marker A, see Figure 1), on the ultrasound probe (Marker B), and on the medial epicondyle of the femur (Marker C), and captured with a four-camera near-infrared VICON® motion capture system (V612, Oxford Metrics Ltd, UK). The tendon length was calculated as the distance between Marker A (= insertion of Achilles tendon) and Marker B plus the distance from Marker B to the muscle-tendon junction (measured with ultrasound). Moreover, muscle length was calculated as the distance between Marker C (= origin of GM) and Marker B minus the distance from Marker B to the muscle-tendon junction. Tendon and muscle lengths were assessed at the end RoM of the pre and post assessment, respectively.

#### Calculation of muscle/tendon force, passive muscle/tendon stiffness, active tendon stiffness, and muscle-tendon stiffness

The muscle force of the GM was estimated by multiplying the measured torque by the relative contribution of the physiological cross-sectional area (18%) of the GM within the plantar flexor muscles (Kubo et al., 2002; Mahieu et al., 2009), and dividing by the moment arm of the triceps surae muscle, which was individually measured by tape measure as the distance between the malleolus lateralis and the Achilles tendon at rest at neutral ankle position (90°, [Konrad & Tilp, 2014]). The mean value of the moment arm was 4.4 cm, with a range of 3.5–5.0 cm.

Active tendon stiffness was calculated as the change in the active force divided by the change of the related tendon length during the MVC measurements over a range of force of 50–90% of MVC (Kay et al., 2015) at 10° plantar flexion. Passive tendon stiffness, muscle stiffness, and muscle-tendon stiffness were calculated as the change in passive force produced at the last 10° up to maximum dorsiflexion divided by the change of the related tendon length, muscle length, and joint angle, respectively. In accordance with Magnusson et al. (1997), the stretching maximum of the pre-test was also taken in the post-test to allow a comparison.

### Stretching exercise

The stretching exercise was undertaken with the dynamometer, with the starting point at neutral ankle position (90°). The subjects were asked to regulate the motor of the dynamometer with a remote control and a maximum angular velocity of 5°/s to get into a dorsiflexion (stretching) position corresponding to the end RoM, with the help of visual feedback. This position was held for 60 s. This procedure was repeated four times, resulting in a total stretch period of 300 s. Between the stretches, the dynamometer moved the ankle into neutral position and back again into the stretching position at 5°/s. The breaks in between the stretches lasted around 20 s. Subjects were asked to be fully relaxed during the stretching exercise.

### Statistical analyses

SPSS (version 20.0, SPSS Inc., Chicago, Illinois) was used for all the statistical analyses. To determine the inter-rater reliability of the muscle-tendon displacement measurements, intraclass correlation coefficients (ICCs) were used. A Shapiro-Wilk test was used to verify the normal distribution of all the variables. To confirm homogeneity of the baseline characteristics of all four groups (0min_post, 5min_post, 10min_post, C), a one-way repeated-measures ANOVA test (normally distributed data) or a Friedman test (other data) was performed. Subsequently, if the data were normally distributed, we performed a two-way repeated-measures ANOVA test (factors: time [pre vs. post] and rest duration [0min_post, 5min_post, 10min_post, C]). Otherwise, we performed a Friedman test to test for differences between the conditions. If the ANOVA or Friedman test was significant, we performed a t-test or a Wilcoxon test to identify the location of the significant differences. An alpha level of *P* = 0.05 was defined for the statistical significance of all the tests.

## Results

### Measurement quality

The mean ICCs of the inter-rater tests of the ultrasound video analysis were 0.98, 0.98, and 0.97 for the muscle-tendon junction displacement during the RoM, PRT, and MVC measurements, respectively.

### Range of motion (RoM) and the related structural muscle-tendon parameters

There was a significant overall effect in RoM seen in the Friedman test (*P* < 0.01; *χ*^2^ = 32.25) and a significant time effect in muscle elongation seen in the ANOVA test (*P* = 0.01, *F* = 10.2, *η*^2^ = 0.48). No group effect (*P* = 0.35, *F* = 1.2, *η*^2^ = 0.29) or interaction effect (*P* = 0.30, *F* = 1.3, *η*^2^ = 0.30) was observed in muscle elongation. The pairwise comparison showed a significant increase in RoM, immediately, 5, and 10 min after the stretching. Moreover, the muscle was significantly elongated at the end RoM when comparing pre- and post-measurements (5min_post + 12.1%; 10min_post + 20.0%). There were small (but insignificant) changes in tendon elongation (5min_post + 2.4%; 10min_post −11.4%). No changes were detected in the control condition (see Table I).

**Table I.** Results of the RoM assessment, including the parameters of RoM, muscle elongation, and tendon elongation.

Rest duration	RoM (°)#	Muscle elongation (mm)§	Tendon elongation (mm)
0min_post			
Pre	29.6 ± 6.8	14.2 ± 3.8	7.8 ± 4.2
Post	34.5 ± 7.8*	16.0 ± 3.4*	9.4 ± 4.5
5min_post			
Pre	30.6 ± 6.8	14.0 ± 4.0	8.2 ± 4.5
Post	34.1 ± 8.5*	15.7 ± 4.0*	8.4 ± 4.3
10min_post			
Pre	29.9 ± 6.1	14.0 ± 3.2	7.9 ± 3.1
Post	32.8 ± 6.1*	16.8 ± 3.3*	7.0 ± 2.9
Control Condition			
Pre	30.7 ± 6.7	13.3 ± 3.3	9.0 ± 4.6
Post	30.1 ± 6.9	14.2 ± 3.8	8.2 ± 4.0

§ = significant time effect (ANOVA). # = overall significant effect (Friedman test), * = significant difference comparing pre- and post-measurements. Mean ± SD.

### Passive resistive torque (PRT) and the related structural muscle-tendon parameters

The ANOVA test showed a significant time effect for PRT (*P* = 0.02, *F* = 10.1, *η*^2^ = 0.59; with no group effect *P* = 0.88, *F* = 0.2, *η*^2^ = 0.11; or interaction effect *P* = 0.39, *F* = 1.2, *η*^2^ = 0.42), muscle-tendon stiffness (*P* = 0.03, *F* = 8.15, *η*^2^ = 0.54; with no group effect *P* = 0.76, *F* = 0.39, *η*^2^ = 0.19; or interaction effect *P* = 0.43, *F* = 1.1, *η*^2^ = 0.39), and muscle stiffness (*P* = 0.03; *F* = 7.97, *η*^2^ = 0.53; with no group effect *P* = 0.95, *F* = 0.1, *η*^2^ = 0.06; or interaction effect *P* = 0.48, *F* = 0.9, *η*^2^ = 0.37). The pairwise comparison revealed a significant decrease in PRT, muscle-tendon stiffness, and muscle stiffness immediately after the stretching. This decrease remained significant in PRT and muscle stiffness at 5 min after the stretching. No changes were observed at passive tendon stiffness, at 10 min after the stretching, and in the control condition (see Table II).

**Table II.** Results of the passive assessment, including the parameters of PRT, muscle-tendon stiffness, muscle stiffness, and (passive) tendon stiffness.

		Muscle-tendon	Muscle	Passive Tendon
Rest duration	PRT (Nm)§	Stiffness (Nm/°)§	Stiffness (N/mm)§	Stiffness (N/mm)
0min_post				
Pre	27.9 ± 12.6	1.6 ± 0.7	17.9 ± 9.3	31.3 ± 25.5
Post	21.9 ± 9.6*	1.2 ± 0.5*	12.6 ± 5.2*	19.6 ± 13.7
5min_post				
Pre	27.8 ± 9.9	1.5 ± 0.5	17.9 ± 6.6	33.4 ± 31.9
Post	25.8 ± 9.3*	1.4 ± 0.5	13.4 ± 5.1*	23.0 ± 13.1
10min_post				
Pre	30.0 ± 13.5	1.7 ± 0.8	19.3 ± 11.4	33.1 ± 23.7
Post	25.4 ± 11.0	1.4 ± 0.6	13.1 ± 6.6	22.6 ± 10.9
Control condition				
Pre	27.7 ± 9.3	1.5 ± 0.6	18.0 ± 7.5	25.8 ± 17.1
Post	27.1 ± 10.2	1.4 ± 0.6	16.5 ± 6.3	33.4 ± 14.9

§ = significant time effect (ANOVA), * = significant difference comparing pre- and post-measurements. Mean ± SD.

### Maximum voluntary contraction (MVC) and active tendon stiffness

The ANOVA test also showed a significant time effect in MVC (*P* < 0.01, *F* = 13.93, *η*^2^ = 0.52; with a significant group effect *P* < 0.01, *F* = 7.5, *η*^2^ = 0.67; but no interaction effect *P* = 0.1, *F* = 2.7, *η*^2^ = 0.42). The pairwise comparison revealed that there was a significant decrease in MVC, immediately, 5, and 10 min after the stretching. No changes were detected in active tendon stiffness or in the control condition (see Table III).

**Table III.** Results of the active assessment, including the parameters of MVC and active tendon stiffness.

		Active tendon
Rest duration	MVC (Nm)§	Stiffness (Nm/°)
0min_post		
Pre	85.8 ± 28.4	35.5 ± 19.5
Post	72.7 ± 27.0*	24.3 ± 12.2
5min_post		
Pre	94.3 ± 31.4	34.0 ± 10.6
Post	89.3 ± 30.8*	29.4 ± 14.6
10min_post		
Pre	96.1 ± 24.6	30.0 ± 13.8
Post	89.6 ± 27.0*	31.2 ± 16.1
Control condition		
Pre	90.8 ± 27.8	34.6 ± 19.3
Post	92.5 ± 28.8	33.7 ± 17.5

§ = significant time effect (ANOVA), * = significant difference comparing pre- and post-measurements. Mean ± SD.

## Discussion

The purpose of this study was to investigate the time course (immediately after stretching = 0min_post, 5 min after stretching = 5min_post, and 10 min after stretching = 10min_post) of possible changes of the MTU function and mechanical properties of the plantar flexor muscles following a 5 × 60 s stretching exercise. As expected, we found an increase in RoM and a decrease in MVC in the three intervention groups, which lasted for at least 10 min. Moreover, muscle stiffness decreased but returned to baseline values between 5 and 10 min after the stretching. No effects were found on the tendon tissue properties at any instant following the stretching.

Similar to previous studies of a single static stretching exercise (Kato et al., 2010; Konrad, Budini, et al., 2017; Konrad, Stafilidis, et al., 2017), RoM was found to be increased in the present study immediately after the stretching. The increase in RoM after a 5-min and 10-min break following stretching is in accordance with the study of Mizuno et al. (2013b), who reported that the retention time of the RoM following a 5-min stretching exercise is between 30 and 60 min. In contrast, Ryan et al. (2008b) found that RoM returned to baseline after 10 min, following 2, 4, and 8 min of stretching. A possible explanation for why the RoM returned to baseline faster than in the study of Mizuno et al. (2013b) and the present study might be the duration of the single stretching bouts. While Ryan et al. (2008) stretched in 30 s bouts (i.e. 4×, 8×, and 16× for 30 s for the 2, 4, and 8-min protocols, respectively), subjects in the study of Mizuno et al. (2013b) and the present study stretched for 60 s per stretching bout (5 × 60 s). Although the overall stretching time was the same in the different studies, the increased number of breaks between the single stretching bouts could have led to a decrease in stretching intensity (i.e. Freitas et al., 2015).

PRT was decreased immediately after 5 × 60 s of static stretching, which is in agreement with several previous studies (Kay & Blazevich, 2009; Konrad, Budini, et al., 2017; Konrad, Stafilidis, et al., 2017; Nakamura et al., 2013). This is also in accordance with the results obtained by Kay and Blazevich (2009), who reported a decrease in passive joint torque immediately after the stretching, which was recovered after 30 min. The design of the present study allowed us to specify that the recovery of the decrease in PRT occurred between 5 and 10 min after the stretching. Concerning overall muscle-tendon stiffness (which is sometimes referred to as “joint stiffness”, e.g. in Kato et al., 2010), we observed a significant decrease immediately after the stretching, but only a tendency of a decrease at 5 min (*P* = 0.06; effect size = 0.56) and 10 min (*P* = 0.07; effect size = 0.53) post-stretching. Decreased muscle-tendon stiffness between 10 and 20 min after 4 and 8 min of stretching was shown by Ryan et al. (2008a). The discrepancies between the study of Ryan et al. (2008a) and our study might be explained by the different stretching intensities used. Whereas Ryan et al. (2008a) stretched at constant torque, i.e. torque was kept constant during the stretching, accompanied with increased joint angle, we performed a protocol with constant joint angle during the stretching, which was probably accompanied by decreasing torque. Previous studies have shown that constant-torque stretching leads to a greater decrease in muscle-tendon stiffness than constant-angle stretching (Cabido et al., 2014). A further explanation for these differences might be found in the different methods used. Ryan et al. (2008a) undertook the post-stretching measurements at 0, 10, 20, and 30 min on the same day, while we undertook the post-stretching measurements (0, 5, 10 min) on separate days. As in our study, Ryan et al. (2008a) performed passive rotation of the ankle joint to measure muscle-tendon stiffness. We believe that such repeated stretches during the test session might affect muscle-tendon stiffness, in a similar way to the intervention.

Concerning maximal isometric contraction movements following a single static stretching exercise, several studies have reported no detrimental effect on maximum performance (Konrad, Budini, et al., 2017; Konrad, Stafilidis, et al., 2017; Kubo et al., 2001; Stafilidis & Tilp, 2015); however, others have showed decreased performance (Herda, Cramer, Ryan, McHugh, & Stout, 2008; Marek et al., 2005) following a single static stretching exercise. These controversial results could possibly be explained by the differences in overall stretch duration, as reported in the review by Kay and Blazevich (2012), who pointed out that stretching for 60 s or longer might induce a detrimental effect on maximum performance. Reid et al. (2018) demonstrated that an additional aerobic activity (dynamic activity and dynamic stretching) to a static stretching exercise up to 60 s even can have a beneficial effect on maximum performance, while 120 s of static stretching (including aerobics) lead to a decrease in performance. As expected, the 5 × 60 s of stretching applied in the present study resulted in a detrimental effect on maximum isometric torque (MVC) immediately after the stretching. In addition, we observed that the recovery time of the MVC is more than 10 min, after 5 × 60 s of static stretching. Therefore, we would not recommend such a large load of static stretching (5 × 60 s) before a competition or training where maximum force is essential.

In addition to the parameters of the muscle-tendon function (RoM, PRT, MVC, and muscle-tendon stiffness), we also investigated the effect of 5 × 60 s of stretching on the muscle and tendon structure separately. The parameters assessed were muscle- and tendon extensibility, muscle stiffness, passive tendon stiffness (measured when MTU is passively stretched), and active tendon stiffness (measured during MVC). As reported in previous studies (Kay & Blazevich, 2009; Kay et al., 2015; Konrad, Budini, et al., 2017; Konrad, Stafilidis, et al., 2017), we observed a decrease in muscle stiffness, but not in tendon stiffness (neither passive nor active), following a single static stretching exercise. However, others have reported a decrease in tendon stiffness (Kato et al., 2010 (passive); Kubo et al., 2001 (active)) with no changes in muscle stiffness (Kato et al., 2010) following a single static stretch. Possible reasons for these controversial results might be found in the different stretch durations (10 min in Kubo et al. (2001); 20 min in Kato et al. (2010)), which we previously discussed in Konrad, Stafilidis et al. (2017). According to the results of the present study, decreased muscle stiffness returned to baseline between 5 and 10 min. This is in accordance, to some extent, with the studies of Mizuno et al. (2013a) and Kay and Blazevich (2009). Mizuno et al. (2013a) found a larger displacement of the muscle-tendon junction (indicating reduced muscle stiffness, assuming similar passive torque) at 15° dorsiflexion angle (but not at 5° or 10°) after 5-min rest following a 5-min stretch. However, this effect vanished 10 min after the stretching, which was similar to the present study. Similarly, Kay and Blazevich (2009) reported decreased muscle stiffness immediately, but not after 30-min rest, following a 3-min stretch. Assuming that the decrease in muscle stiffness is based on an increase in resting sarcomere length induced by the stretching (e.g. by lengthening titin) (Gajdosik, 2002), these results could be an effect of the restored sarcomere lengths between 5 and 10 min after the stretching. An increase in sarcomere length could also explain the decrease in MVC. Interestingly, MVC was still reduced 10 min after stretching, although muscle stiffness apparently returned to baseline values. However, a closer look at the data 10 min after stretching revealed a tendency of reduced muscle stiffness (−6.2 N/mm; *P* = 0.1), indicating that reduced muscle stiffness might be related to reduced MVC. However, this could not be confirmed by a correlation analysis between the delta values of MVC and muscle stiffness 10 min after the stretching (*P* > 0.05).

A further possible mechanism which explains functional changes (increased RoM and decreased MVC 10 min after the stretching) and the absence of mechanical changes (i.e. muscle stiffness) might be an increased stretch tolerance and perception of pain (Magnusson et al., 1996). Our data support this hypothesis since PRT at the end RoM (pre and post) was significantly increased at all three time points (0min_post + 37.5%, 5min_post + 44.3%, and 10min_post + 32.9%).

Since we measured muscle and tendon stiffness with force-elongation curves we might have neglected other possible mechanism which are responsible for mechanical changes like synergist muscles, joint capsules, nerves, skin, and fasciae (Weppler & Magnusson, 2010). Furthermore, the seated position during the experiments might have stretched the sciatic nerve during the static stretching exercise and therefore affected the RoM (Andrade et al., 2018). Thus, changes in sciatic nerve might also explain the changes in the function and the absence of changes in the measured mechanical parameters.

## Perspectives

We conclude that a single static stretching exercise over 5 × 60 s increases the RoM and decreases MVC for at least 10 min. However, these changes can only be partially explained by more compliant muscle tissue within the first 5 min after the stretching. Hence, increased RoM and decreased MVC might additionally be associated with increased stretch tolerance and changes in the sciatic nerve.
